# 

**Published:** 1902-03

**Authors:** 


					﻿*c
O-
LLl
QE
O-
LlJ
G9
*C
1“
CO
o_
■
1
o
Id
O
ID
CM
T"
o
2
o’
o
2
0)
o>
■
co
0)
I
K
0)
i
(0
0)
2
u.
a
co
ULI
o_
C5
CJ
aa
LEADS VETERINARY JOURNALISM IN AMERICA.'
Vol. XXIII.
MARCH, 1902.
No. 3
PUBLISHED MONTHLY AT $3 A YEAR. SINGLE COPIES, 30 CENTS.
FOREIGN SUBSCRIPTIONS /3.50 PER YEAR.
THE JOURNAL
OF
Comparative Medicine
AND
VETERINARY^ARCHIVES
EDITED AND PUBLISHED BY
W. HORACE HOSKINS, D.V.S.,
00
o
c
z
o
o
o
2
fi
(D
o
"Tl
(0
o
u
Fl
u
Fl
>
□
<
“n
O
00
□
Fl
r
<
Fl
-<
ALL COMMUNICATIONS AND PAYMENTS SHOULD BE ADDRESSED TO
Dr. W. Horace Hoskins, Managing Editor, 3452 Ludlow St., Philadelphia, Pa.
Copies may be had on order of all news-dealers at home and abroad.
AN ADVERTISING MEDIUM UNEQUALLED IN THE VETERINARY FIELD
				

